# Catalog of 2017 Thunderstorm Ground Enhancement (TGE) events observed on Aragats

**DOI:** 10.1038/s41598-019-42786-7

**Published:** 2019-04-18

**Authors:** A. Chilingarian, H. Mkrtchyan, G. Karapetyan, S. Chilingaryan, B. Sargsyan, A. Arestakesyan

**Affiliations:** 1 Alikhanyan National Lab (Yerevan Physics Institute), Alikhanyan Brothers 2, Yerevan, 0036 Armenia; 2 National Research Nuclear University MEPhI (Moscow Engineering Physics Institute), Moscow, 115409 Russian Federation; 3 Space Research Institute of RAS, Moscow, Russia

**Keywords:** Atmospheric dynamics, Plasma-based accelerators

## Abstract

The natural electron accelerator in the clouds above Aragats high-altitude research station in Armenia operates continuously in 2017 providing more than 100 Thunderstorm Ground enhancements (TGEs). Most important discovery based on analysis of 2017 data is observation and detailed description of the long-lasting TGEs. We present TGE catalog for 2 broad classes according to presence or absence of the high-energy particles. In the catalog was summarized several key parameters of the TGEs and related meteorological and atmospheric discharge observations. The statistical analysis of the data collected in tables reveals the months when TGEs are more frequent, the daytime when TGEs mostly occurred, the mean distance to lightning flash that terminates TGE and many other interesting relations. Separately was discussed the sharp count rate decline and following removal of high-energy particles from the TGE flux after a lightning flash. ADEI multivariate visualization and statistical analysis platform make analytical work on sophisticated problems rather easy; one can try and test many hypotheses very fast and come to a definite conclusion allowing crosscheck and validation

## Introduction

In recent years, the interest in using cosmic rays for obtaining information on atmospheric and extra-atmospheric processes is rapidly growing. Cosmic rays are modulated by the solar bursts and can be used as messengers carrying information on upcoming space storms. Recently it was discovered that particle fluxes also carry information on the parameters of atmosphere, primarily on very difficult to measure atmospheric electricity. Fluxes of gamma rays and electrons carry information on the net potential in the atmosphere related to emerging positive and negative charged layers in the thundercloud. Cosmic ray electrons entering electric field accelerates either in direction to the surface or - into open space. Bursts of gamma rays detected in the space are called Terrestrial Gamma Flashes^1–3^, in the atmosphere - gamma glows^4–7^, on the earth’s surface - Thunderstorm Ground Enhancements^8–14^. Along with gamma rays and electrons on the earth’s surface also neutrons were detected^15–17^. The duration of particle fluxes varies from microseconds to hours^18,19^. To explain gamma ray bursts reaching orbiting gamma ray observatories, models were presented suggesting a new source of seed electrons from very large electric fields in the vicinity of lightning leaders^20^.

Runaway Breakdown (RB^21^), or, as it termed in many publication, Relativistic Runaway Electron Avalanche (RREA^22,23^), is the main mechanism more or less satisfactory explaining electron accelerators operated in the clouds. Recently the RB/RREA mechanism was supplemented by a new source of the electrons, accelerated in the streamer tips of a developing lightning leader^24^. Electrons are accelerated up to energies of ~70 keV and then runaway^25^. In any case, the lower dipole between the main negative charge and its mirror on surface or/and lower positive charge layer (LPCR) in the bottom of cloud supports the downward electron acceleration; the electric field strength in the lower part of the thundercloud is also crucial for the lightning flash development. However, due to the difficulties of *in situ* measurements of intracloud electric fields, both electron acceleration and lightning initiation are not well understood until now. Thunderstorm is an important part of the global electrical circuit (GEC), along with ionosphere, clear air, conducting earth, thunderstorms, and lightning^26^. The atmospheric electric fields and atmospheric discharges in last decades were intensively investigated using radars, 3D lightning mapping arrays, worldwide lightning location networks, observations of wideband electric field waveforms, and by the wideband and narrowband VHF interferometer systems, and sensors measuring near-surface electric field. In^27^ was established that large LPCR precludes negative cloud-to-ground lightning flashes (-CG), and only in the end of the storm –CG could be triggered. Nag and Rakov describe various scenarios of atmospheric discharges dependent on the maturity of LPCR^28^. In^29^ was noticed that -CG lightning frequently started as an inverted-polarity intracloud discharge that partly deactivated the lower positive charge so that a cavity in the LPCR was formed that assists a -CG discharge. In turn, the intense TGE can provide enough ionization to facilitate intracloud discharge and usually discharges occurred just after the maximum of particle flux^30^. Thus, lightning flashes and TGEs are interconnected phenomena and should be studied comprehensibly. H.Tsuchiya in^31^ suggested that warm winds moved from the sea, originate winter thunderstorms in Japan with short- lived tripole structures appeared, which accelerate CR electrons toward the bottom positive layer. Chilingarian and Mkrtchyan in^32^ mentioned the role of LPCR in the TGE initiation. In^33^, the role of the main negative charge in the cloud and its mirror image on the ground for the downward electron acceleration was established. This field is influenced by other charges in the cloud and can be locally enhanced by the LPCR. In^34^ were considered different scenarios of lower dipole development by engaging TGE and near-surface electric field observations. Thus, there are different scenarios of TGE initiation and lightning occurrence. However, they are dependent on each other and should be analyzed together for scrutinizing the structure and evolution of the lower dipole.

In our recent papers^35,36^ we outline and classify TGE subsample abruptly terminated by the lightning flash. A large share of TGEs abruptly terminated by lightning flashes is due to -CG flashes and normal-polarity intracloud flashes. A smaller portion of TGEs that were terminated by lightning discharges were related to inverted-polarity intracloud flashes (-IC) and hybrid flashes (inverted-polarity -ICs followed by -CGs).

On Mt. Aragats the networks of detectors registering electrons, muons, gamma rays, and neutrons and providing important information on various geophysical processes was operated since 1943^37^. A huge amount of registered time series should be processed and identified near on-line for forecasting and alert issuing, as well as for the reports and scientific papers. To support researcher in data mining and finding “new physics” a multivariate visualization platform should be supplemented with tools of the statistical analysis (histograms, moments, correlations, comparisons); figure preparation; archiving, i.e. with a data exploration system. We connect the online stream of “big” data from ASEC to an exploration system^38^ that helps researchers in understanding solar-terrestrial connections, solar modulation effects as well as in understanding high-energy phenomena in the atmosphere. Time series from different domains are joining for multivariate correlation analysis and physical inference.

## Results

### Long Lasting Low Energy Thunderstorm Ground Enhancements (LLL TGE)

**Figure 1:**
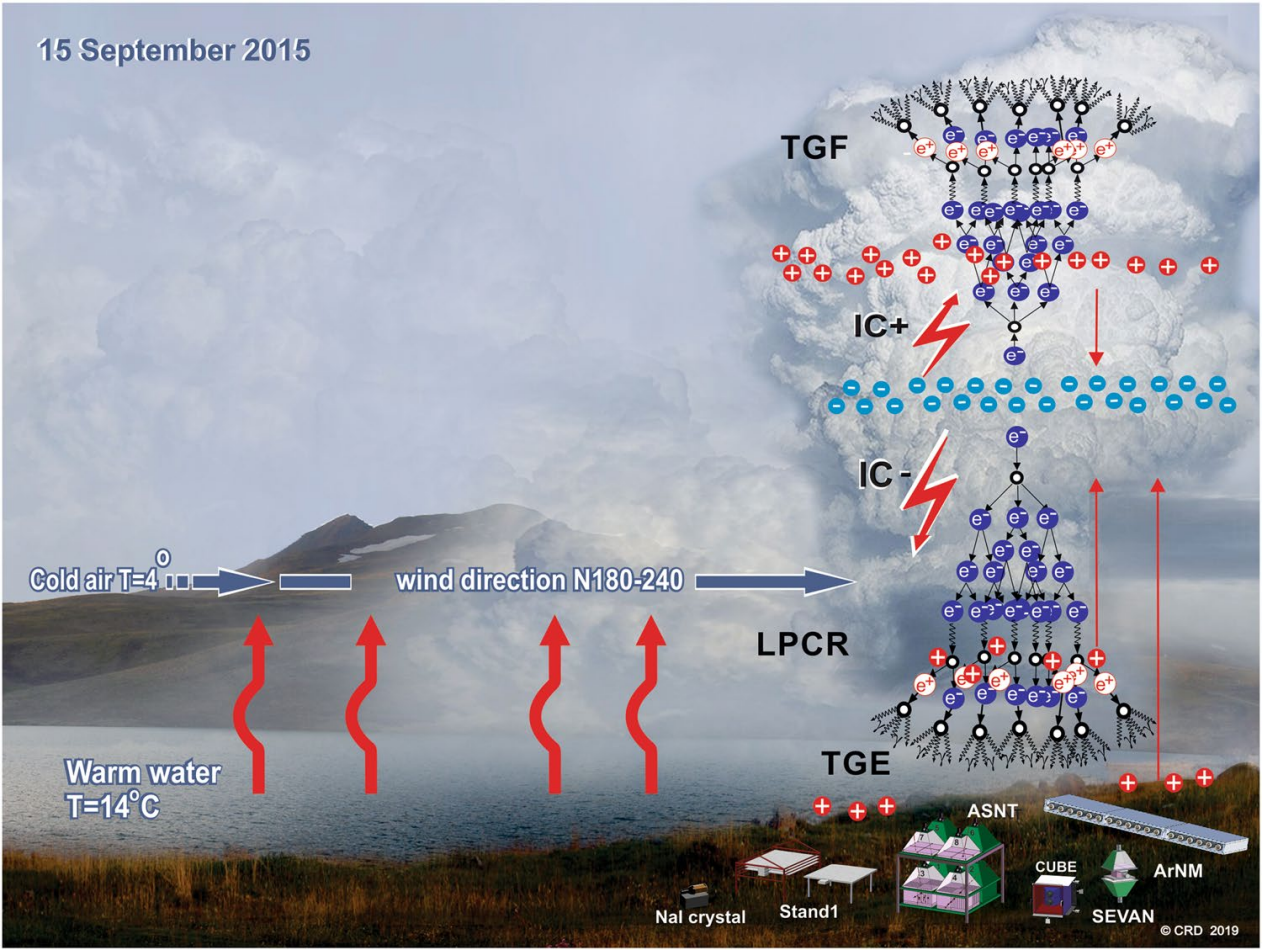
The lake-effect: cloud origination due to an updraft of the moisture brought by wind from the warm lake surface.

In 2017 Aragats facilities register more than 100 TGEs, most of them originate in cumulonimbus clouds due to charge separation triggered by the moisture updraft of orographic and lake effects, see Fig. 1.

In the right side of Fig. 1, we show particle avalanche developed in the lower part of the thundercloud shaped by the main negative charged region and its mirror on the earth’s surface (long red arrow) and by the same negative charged region with LPCR (short red arrow).

**Figure 2:**
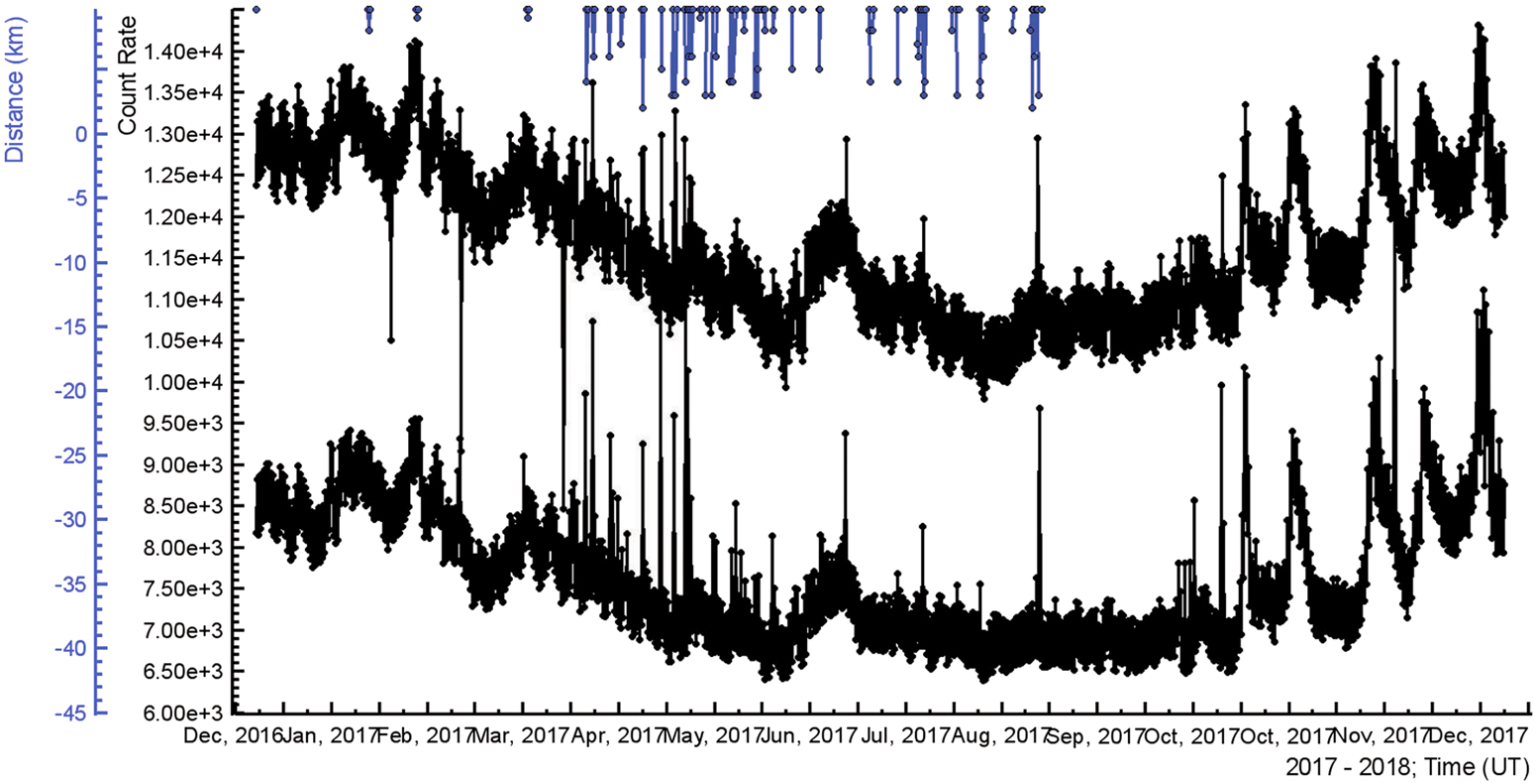
Distances to lightning discharges (indicated by the line segment ending in the top) and time series of the count rates: middle curve –upper scintillator of the STAND3 detector; bottom – 20 cm thick scintillator of the CUBE detector.

In Fig. 2 we present the particle detector counts and occurrences of lightning flashes inside a radius of 10 km. The bottom curve was measured by 3 cm thick one m^2^ area plastic scintillator; the upper curve – by 20 cm thick 0.25 m^2^ area plastic scintillator. The energy threshold of the first scintillator is ~3 MeV, of the second ~6 MeV. In the top of Figure, the distance to lightning discharge is shown. The total number of registered lightning with distances to the detector site less than 10.5 km was 1450; thus, the frequency of lightning flashes nearby Aragats station was in 2017 ~5 per km^2^ per year.

We can notice in the rather coherent time series (correlation of time series is ~ 98%) multiple small and large coincided surges that are more frequent in the spring. In the next Figures, we demonstrate zoomed versions of these surges, i.e. TGEs lasting from a minute to several hours.

**Figure 3:**
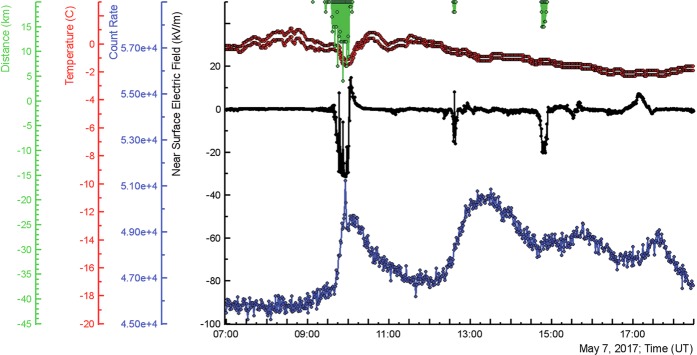
In the bottom –count rates registered by NaI crystal (energy threshold 0.3 MeV); in the middle - disturbances the electric field; on the top - outside temperature, dew point and distance to the lightning flash.

In Fig. 3 we demonstrate one of TGE events, occurred on 7 May 2017, the month of the maximal thunderstorm and TGE activity. The one-minute time series are measured by a large (12 × 12 × 25 cm) NaI crystal located under the roof of the experimental hall; near surface electric fields and distances to discharges were measured by electric mills; the outside temperature and dew point used for calculation of the distance to the cloud base were measured by the Davis weather station. The pattern of TGE is rather complicated, demonstrated several peaks and deeps directly related to the disturbances of the near surface electric field (superposition of the electric fields induced by several charged layers in the thundercloud). The first peak (from the left) started at ~9:00 UT and prolonged to ~11:30 demonstrates sharp surge at 9:57. The sharp particle outburst occurs when the electric field was in the deep negative domain (~ −30 kV/m) for ~20 minutes from 9:40 to 10:00. The nearest lightning flash was registered on 3.0 km from detector site. The cloud base was ~50 m above surface, relative humidity ~96%.

The second peak started at 12:05 is much smoother; the electric field again was in negative domain ~ −14 kV/m for 5 minutes (12:35–12:40).

**Figure 4:**
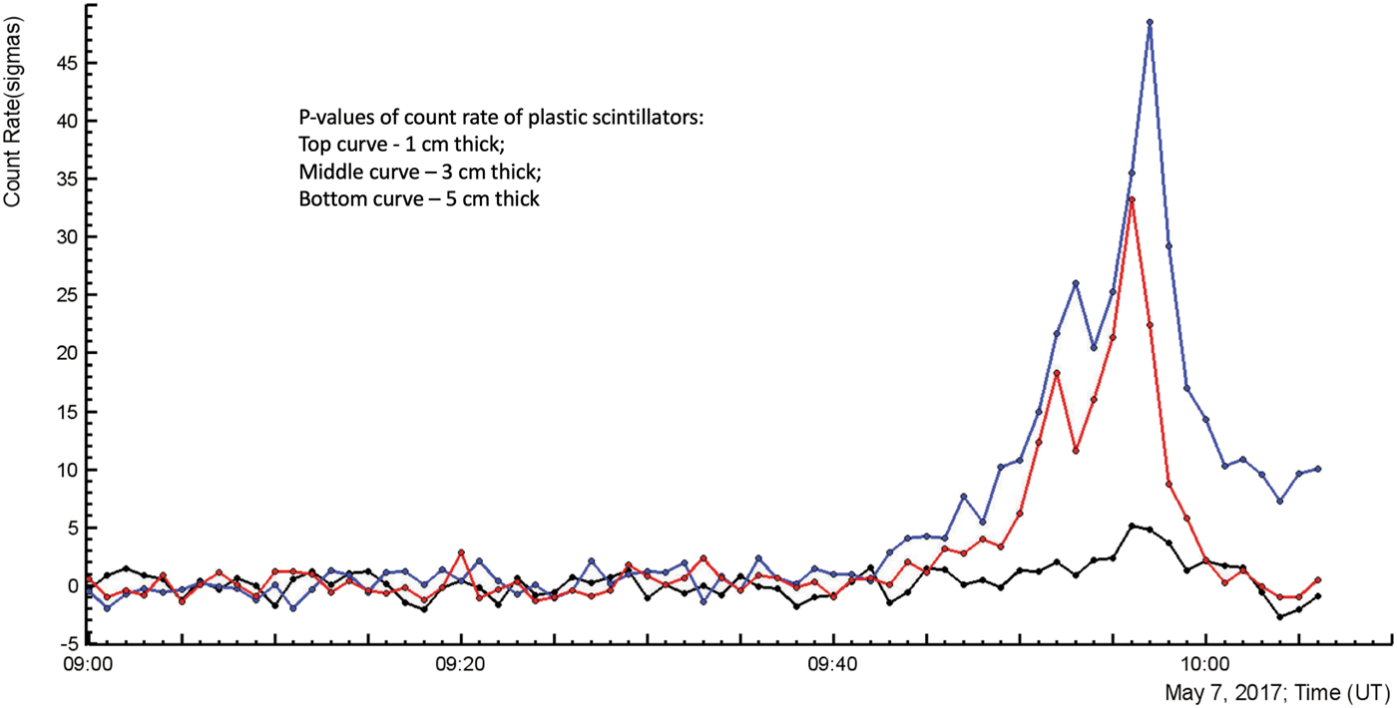
Time series (in the number of σ) of 1, 3 and 5 cm thick 1 m^2^ area plastic scintillators. Energy threshold correspondingly ~0.8, 3 and 5 MeV.

In Fig. 4 we show the time series of p-values of measured peaks (i.e. how many standard deviations from mean values are contained in the peak).

We show the time-series of particle count rates in p-values for comparative purposes only. Comparison of the detectors with different sizes and different energy threshold in absolute counts make no sense because most of structures will be smoothed if measurements will be scaled according to largest count rate. No structures for detectors with small count rates will be seen. However, showing time series in the p-values, as we see in Fig. 4., reveals the structures ever for scintillators with large energy threshold shown along with detectors with low energy threshold (the absolute count rates of both are drastically different). The mean value of the time-series is calculated with one-third of the time shown in the picture (the left third of the whole X-axes).

In Fig. 4 we show one-minute count rates of three plastic scintillators with different energy thresholds. The 1-cm thick scintillator located outdoors has the lowest energy threshold (~0.7 MeV), and correspondingly – the highest p-value of 47σ. The lowest p-value of 5.5 σ shows 5 cm thick scintillator located in the MAKET building (energy threshold ~7 MeV).

**Figure 5:**
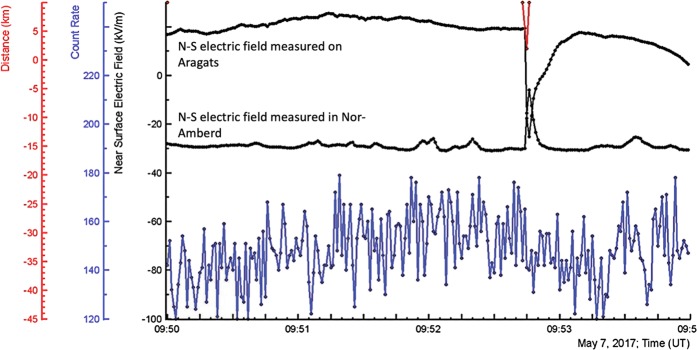
Distance to lightning flash; disturbances of near-surface electric field measured in Nor Amberd and Aragats (polarity inversion is apparent); one-second time series of the outdoor plastic scintillator.

In Fig. 4 as well are seen 2 nearby peaks in the TGE. 1-minute time series cannot provide all details for exploring emerging structures in the particle flux; therefore, in Fig. 5 we show the one-second time series of the same-type 1-cm thick plastic scintillator, along with disturbances of electric field measured at two high mountain research stations (distance between stations ~ 13 km).

Rise of count rate started at 9:50, followed by a sharp decrease related to the nearby lightning flash occurred at 9:52:45. The polarity of lightning at Aragats was negative, increasing from −30 kV/m to −5 kV/m (amplitude 25 kV/m); polarity of lightning in Nor Amberd was positive,

decreasing from 20 kV/m down to −30 kV/m (amplitude 50 kV/m). Thus, polarity was reversed in Nor Amberd; we identify this kind of lightning flashes as a normal-polarity IC. Such a type of lightning flashes is observed at Aragats quite often. It can be considered as an evidence of mature LPCR, providing large potential drop for electron acceleration and preventing lightning leader to reach the ground. An example of such a flash (not associated with TGE termination) was shown in Figs 8 and 9 of Chilingarian *et al*., 2017. Due to the low height of the cloud, the reversal distance is small, and ~13 km between Aragats and Nor Amberd stations is sufficient to detect apparent polarity reversal.

**Figure 6:**
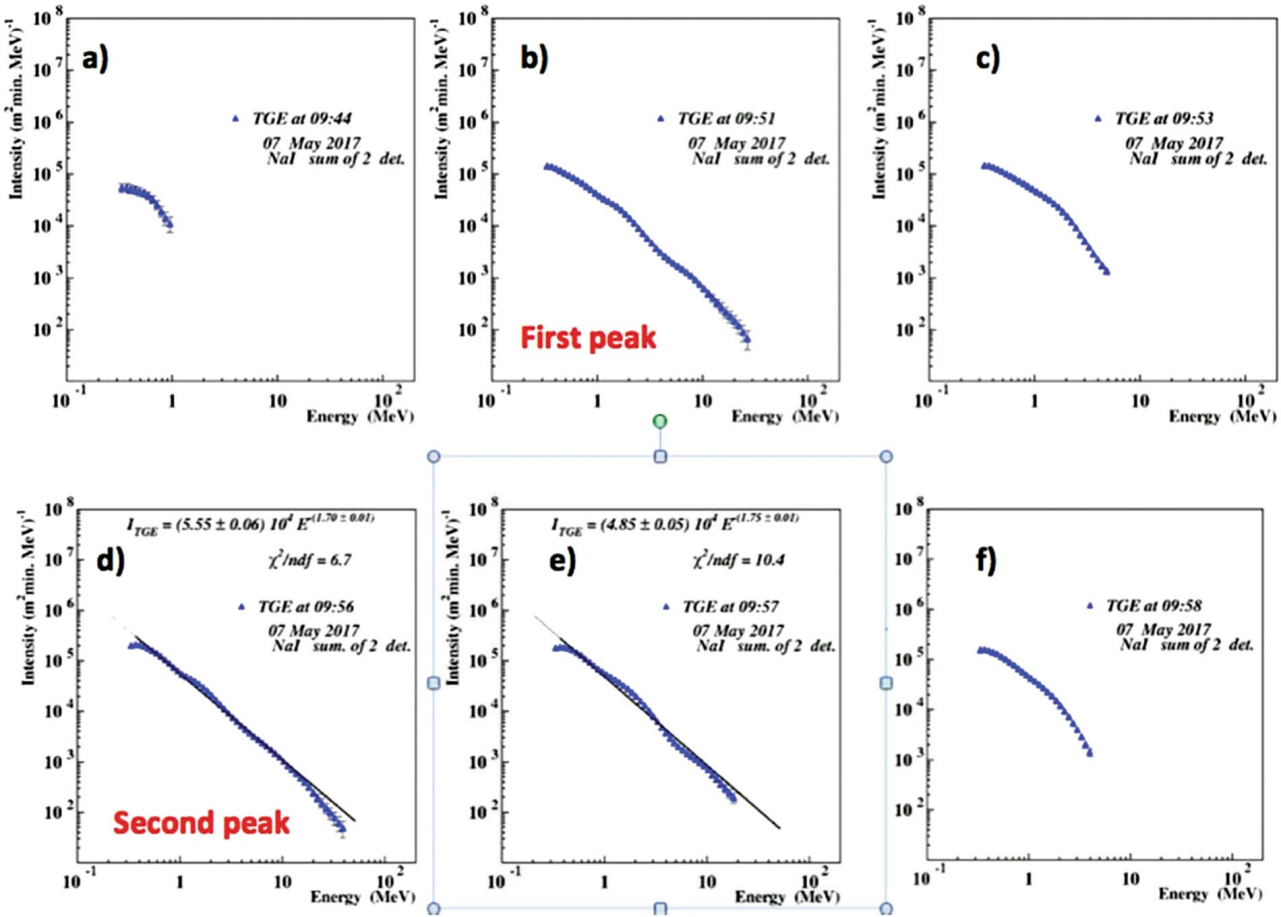
Differential energy spectra of the 7 May TGE measured by the NaI crystals (NN 1 and 2).

To find out the origin of the count rate decline we estimate differential energy spectra with the network of NaI crystals. In Fig. 6 we show differential energy spectra of the gamma ray flux from the start (Fig. 6a) to the first maximum at 9:52 (Fig. 6b) terminated by the lightning flash at 9:52:45; then we show the second maximum at 9:56 (Fig. 6d) decaying at 10:58 (Fig. 6f). From Fig. 6b,c we see that lightning “kills” flux of high-energy particles (HEP). Before lightning flash, the maximal energy reaches 30 MeV (Fig. 6b) and after lightning (Fig. 6c) only 6 MeV. We can see from Fig. 6 that for the smaller peak maximal energy reaches ~30 MeV, and for the second, larger – ~40 MeV. For the NaI crystals maximal achievable energy that can be recovered by the energy release histograms is ~50 MeV. The intensity of higher energies is so small that ever large NaI crystals hardly will detect at least 5 particles in the histogram bins above 50 MeV. Therefore, inherent background fluctuation will not allow reliable energy recovering. Another spectrometer with larger size (Aragats Solar neutron telescope, ASNT) is used for measuring TGE energies up to ~100 MeV.

Another important question in the establishing of long lasting TGE is the radiation of Radon progenies contributing to the TGE flux in the low energy domain (below 3 MeV). Fair-weather (background) low energy gamma ray spectra measured on Aragats is a mixture of the continuous spectrum produced by galactic (and, sometimes, also solar) cosmic rays in interactions with the atmosphere and emission lines of long-lived nonstable nuclides (^238^U, ^235^U, ^232^Th, etc.). The half-life of the radon isotope ^222^Rn is 3.82 days, thus it can be transported to the atmosphere due to the diffusion mechanism. Although we demonstrate that the hypothesis of the precipitation as a source of gamma ray radiation initiated TGE is not valid^39^, it was proposed that Rn-222 can be concentrated in the clouds above Aragats research station and radiation of its daughter isotopes can lead to the observed prolonged low energy part of TGE^40^. To identify the role of the gamma radiation from Radon progenies in the long lasting TGE flux, differential energy spectrum was measured with various spectrometers, including precise 3″ × 3″ NaI(Tl) spectrometer of ORTEC firm (FWHM ~7.7% at 0.6 MeV). Measurements demonstrate that Radon progenies radiation significantly contributes to the “Winter TGEs” in the energy range below 3 MeV^41^. However, both Monte Carlo simulations^42,43^ and measurements of gamma ray flux with large detector setups demonstrate that TGEs are originated in the intracloud electric fields.

### Statistical analysis of TGE events observed in 2017

We present TGE catalog for 2 broad classes according to presence or absence of the high-energy particles (>3 MeV). In Tables 1 and 2 we summaries several key parameters of the TGEs observed in 2017 and related meteorological data. For both Tables, the selection criteria were the selected enhancement (peak) significance not less than 4σ. We put the date of the TGE and time of the occurrence of the largest peak in the first column (the NaI scintillator’s one-minute time series was used for the peak identification); in the second column – occurrence time of the second peaks (if any); In 3–6 columns – the significances of the peaks (in percent/and in number of standard deviations) for particle detectors with different energy thresholds:

- NaI crystal, energy threshold 0.3 MeV;
- NaI crystal with energy threshold 3 MeV;
- The upper plastic scintillator of the STAND1 detector (energy threshold ~ 0.8 MeV);
- The upper scintillator of SEVAN detector, energy threshold 7 MeV.

**Table 1:** TGE events containing High-Energy particles.

Date by NaI_0.3_ UT	Peaks 2nd, 3rd by NaI_0.3_ UT	%/Nσ				∆T (min.)				Dist to flash (km)	Dist. to cloud base (m)	Temp. (°C)	−E (kV/m)	∆E (kV/m)
		E_th_ (NaI) ~0.3 MeV	E_th_ (NaI) ~3 MeV	E_th_ (Stand) ~0.7 MeV	E_th_ (Sevan) ~7 MeV	NaI_0.3_	NaI_3_	STAND	SEVAN					
Apr. 7 14:20	—	7/16	12/7	16/17	3.5/4.4	180	10	180	10	3	134	−3.1	24	65
Apr. 9 11:00	—	12/21	12/7	18/19	5/6	20	25	15	20	11	85	−4.8	22	33
Apr. 9 12:00	—	15/27	19/11	24/21	7/9	50	25	30	20	12	85	−4.1	29	40
Apr. 14 12:00	—	13/22	12/9	17/18	4.5/7.6	150	3	104	3	3	37	−0.6	18	39
Apr. 15 11:52	—	13/22	7/5	15/15	3/4	220	15	180	20	12	36	−0.3	26	28
Apr. 16 15:00	—	8/18	6.4/4	14.5/16	3/4	130	20	130	20	6	85	−3.4	24	39
Apr. 23 21:08	—	10/20.5	9/8	23/26.5	3/4	120	15	140	5	4	49	−1.6	27	73
Apr. 23 23:58	—	5/7	11/6	16/9	3/4	100	10	150	10	7	49	−1.6	42	73
Apr. 24 1:46	—	2.6/4	10.6/8	10.9/6.4	—	10	5	20	—	4	49	−2.2	34.5	52
Apr. 29 11:54	—	15/28.3	27.5/17.4	48.2/47	7.9/9	136	22	134	21	4	37	−0.6	22.8	58
May 3 01:08	—	8.5/10	13.2/10.6	23.8/21.2	5/6.8	170	25	30	27	16	37	0.2	31	36
May 3 13:53	13:59	42.7/48	73.3/39.5	45/38	7.2/8.4	110	4	120	22	3	50	0.4	22.9	70
May 6 12:40	—	11.1/21.5	26/17.9	44/36	8.8/10.8	231	45	220	30	3	98	0.7	33.8	80
May 6 14:00	—	14.4/15	9.5/5.6	27/17	3.8/4.3	95	20	25	10	10	60	0.1	40	54
May 7 9:56	9:53	11.4/21.7	09/10	40/30	4.3/4.8	120	5	120	20	4	50	−1	26	58
May 8 0:0	—	4/6.9	11.3/8.2	25.8/23.1	4.3/5.2	51	21	83	21	7	61	−2.4	21.2	67
May 14 14:48	—	11.7/15.7	14.2/8.9	15.8/16.9	—	100	15	110	—	3	159	1.9	19.5	60
May 19 17:13	—	5.9/5.1	8.3/5	9.8/5.4	3.2/4.2	139	30	112	31	2	159	2.1	23.6	64
May 20 02:25	3:49	11/21	4.5/3.1	18/13.4	3/4.4	180	8	190	15	9	25	1.8	19	24
May 20 7:50	—	10.4/18.6	7.1/4.9	4.7/4.8	—	80	5	85	—	6	37	2	22	36
May 21 14:15	—	6/5	10/6	22/16	4/5.5	170	15	25	20	7	49	−1.2	30	43
May 26 16:49	17:25	2.4/4.7	5.5/3.7	—	3.7/4.7	110	16	—	13	2	159	2.8	29.1	61
May 27 14:25	15:18	1/6.7	7/4	—	3.1/4.2	136	10	—	11	2	49	1.1	29.7	74
June 1 4:23	5:18	8/17	7/4.1	13/16.2	—	106	10	10	—	16	150	−0.3	32.9	58
June 1 08:16	9:06	8.9/15.4	7.5/5.1	10/13	4.1/5.7	155	20	19	19	12	120	0.7	19.6	51
June 15 23:56	—	3/4	4.5/4	—	3/4.5	70	20	—	30	14	135	2.8	17	25
June 21 20:53	—	8.4/17.2	3.4/3.5	06/13.0	2.4/3.8	140	—	138	—	7	219	4.1	9.56	33
June 22 13:52	14:12,14:16	7.5/16.2	15.1/10.1	18.1/23	10.4/14.9	165	42	130	41	7	170	4.4	32.4	54
July 7 13:23	13:44,13:49	6/10	6/4	—	3/5	123	20	—	20	5	280	7.2	15	43
July 15 6:28	6:57, 8:00	23/11	15.6/10.7	—	7/9.6	180	—	—	13	6	195	7.8	16.8	32
July 24 18:40	18:54	13/16	9/6	—	4/6	180	15	—	20	9	220	7	22	51
July 31 16:04	16:09	23/49	16/12	45/41	5.8/8.2	240	6	273	14	8	130	7.4	24	31
Aug. 17 11:00	—	4.9/7.2	5.5/8.8	7/6.1	4.2/5.3	90	8	73	7	5	268	7.6	23.6	42
Aug. 17 18:55	18:57	19.8/46	52.7/32.5	6/5.6	16.4/27	210	6	180	14	2	200	8.4	21	49
Sept. 29 21:52	—	12/22	7/5	12/21	6.1/8.1	120	4	11	18	16	25	0.4	15	25
Oct. 1 5:58	—	8/20	13/9	14/9	7/9.3	60	6	12	13	15	200	2.9	22	44
Oct. 1 20:33	—	16.6/21.8	5/3.5	21.3/13.2	3/5	140	10	135	5	5	85	0.7	26	53
Oct. 2 8:04	—	7/12	11/7	18/15	7/11	60	5	60	4	16	50	−0.7	21	42
Oct. 10 12:18	—	4.9/11	6.5/4.3	9.3/10.8	9.5/13	110	15	85	10	2	85	1.3	14	36
Oct. 10 14:08	14:10	10/13.7	25/20	13/11	12.8/12.4	120	8	155	15	2	150	0.5	22	48
Oct. 10 22:04	22:12	6.3/11	12.5/8	12/16.4	8/10	160	15	160	20	13	37	−0.2	8	22
Nov. 7 7:12	8:00,08:30	7/8.5	6/7.4	—	—	191	188	—	—	19	25	−0.2	10.4	32
Nov. 8 5:30	—	5/5.9	6.2/7.1	3.8/3.5	—	107	120	104	—	21/16	25	−0.1	9.8	16
Nov. 30 3:45	—	7/12	9.5/14.5	51/49	11.1/19	124	150	180	24	14	98	−7.2	5.38	15

**Table 2:** TGE events that do not contain High-Energy particles.

Date by NaI_0.3_ UT	Peaks 2nd, 3rd by NaI_0.3_ UT	%/Nσ				∆T (min.)				Dist to flash (km)	Dist to cloud base (m)	Temp. (°C)	−E (kV/m)	∆E (kV/m)
		E_th_ (NaI) ~0.3 MeV	E_th_ (NaI) ~3 MeV	E_th_ (Stand) ~0.7 MeV	E_th_ (Sevan) ~7 MeV	NaI_0.3_	NaI_3_	STAND	SEVAN					
Apr. 8 14:00		5.2/11	—	6/7.5	—	480	—	310	—	13	85	−4.7	9	22
Apr. 9 21:02		3/4.5	—	—	—	65	—	—	—	12	60	−3.8	24	37
Apr. 17 16:41	—	6.7/7.7	—	12/7.7	—	80	—	100	—	4	85	−2.3	28	43
Apr. 30 13:53		2.9/5.8	—	4.6/4.8	—	160	—	30	—	23	49	0.5	22	22
May. 5 11:25		8.4/12.7	—	—	—	160	—	—	—	4	134	0.5	30	41
May 6 02:10		6.7/10.8	—	8.1/9	—	140	—	300		16	37	−0.7	22	24
May. 6 09:10		21.9/30	—	18.4/18.5	—	160	—	240	—	33	60	0.4	11	19
May 8 13:46		9/10	—	13/12	—	240	—	160	—	5	25	−0.1	7.5	8
May 9 16:26		12/27	—	14/14	—	160	—	120	—	16	60	1.4	14	38
May 10 14:10		2.3/4.4	—	—	—	25	—	—	—	7	160	2.9	11	20
May 10 20:31		3.1/4	—	4/4.6	—	80	—	70	—	20	200	3	7	20
May 10 22:21		8.5/11	—	10/10	—	90	—	100	—	6	170	2.8	11	32
May 12 14:06		7/17.6	—	9.4/12	—	60	—	90	—	3	195	1.6	23.6	62
May 14 8:53	8:56	7.6/13.2	—	7.5/11.3	—	105	—	180	—	24	110	1.5	7.8	14.5
May 14 13:22		4.3/7.9	—	16.9/16.4	3.4/4.5	90	—	70	15	16	85	1.9	24	28
May 15 12:05.		12.3/8.5	—	18.2/19.7	3.7/4.7	278	—	267	32	7	85.4	−0.1	34.7	69.4
May 19 13:48	13:51	4.6/7	—	50/66	—	30	—	12	—	10	120	3.8	21	46
May 19 15:34	15:39	4/4	—	6/3.5	—	56	—	54	—	6	130	2.9	18.5	35
May. 22 14:57		5.1/11.7	5/3.0	37.9/23.4	4.7/6.7	80	10	38	13	5	48.8	−0.1	21.5	42
May 23 07:55		16/30	—	15/13	—	360	—	120	—	6	61	−0.4	16	16
May. 23 13:35	14:17	8.7/12.5	—	8.5/9.5	—	160	—	200	—	—	85	1.4	3	7
May 23 20:26		8.8/16.9	—	—	—	360	—	—	—	23	37	−0.8	6.9	18
May 24 15:18		2.3/4.6	—	—	—	120	—	—	—	10	37	−1.3	12	12
May 27 3:36		11.8/15.4	—	—	—	100	—	—	—	16	37	−0.1	11	15
May 29 5:14		3/6	—	—	—	30	—	—	—	15	120	−0.2	16	22
May 29 16:30		2/4.5	—	4.5/5	—	65	—	60	—	12	310	4	18	50
June 6 17:35		2.7/4	—	5.5/4.7	—	104	—	199	—	3	790	8.1	22.8	43
June 11 15:30.		3.3/6	—	4.2/5.4	—	99	—	132	—	27	350	4.7	3	7
June. 12 15:22	16:30	3.8/7.3	4/3.0	5/5.3	—	131	8	180	—	20	180	4	12.3	32
June 16 10:56		10/12.5	—	7/10	—	200	—	180	—	25	110	2	14	24
June 20 10:23		8/15	—	4/5.4	—	110	—	180	—	27	120	6	5	10
June 20 12:55		7/21	—	5/6.5	—	80	—	60	—	14	300	7	5	21
June 29 09:25		17.2/23.7	—	—	—	300	—	—	—	4	330	8.4	23	49
July 13 10:12		6.9/9.2	—	—	—	210	—	—	—	6	160	9.6	7	20
July 13 13:51		11.8/15.7	—	—	—	110	—	—	—	10	240	7.5	3.5	16
July 13 18:18		11.7/15.5	—	—	—	180	—	—	—	18	120	4.7	5	17
July 14 16:17		3.5/7.3	—	—	—	160	—	—		25	440	9.3	16	16
July 15 16:16	18:00	4.7/4	—	—	—	63	—	—	—	5	232	7.6	18.2	34.5
July 15 18:21		4.8/9.2	—	—	—	160	—	—	—	6	250	7.1	25	46
Aug. 2 9:25		15/10	—	8.2/7.7	—	60	—	150	—	7	500	13	2	15
Aug. 2 10:20		21/14.4	—	—	—	250	—	—	—	10	610	15	7	16
Aug. 10 9:20	10:28	10.1/9.5	—	—	—	180	—	—	—	6	780	12.4	11.9	35
Aug. 11 10:00	11:11	7.7/9	—	4/2.8	—	558	—	75	—	7	561	12.9	4.8	14
Aug. 15 10:34		8/8.4	—	7/5.7	—	40	—	40	—	25	340	10.4	4	8
Aug. 15 11:36	11:49	10.6/11	5/3.6	14/11	—	70	8	60	—	14	270	4	5	31
Aug. 15 12:51		19/20	—	25/20	—	35	—	40	—	7	290	10.7	3.6	16
Aug. 15 13:55		22/24	—	29/23	—	55	—	35	—	12	270	10.2	7	21
Aug. 16 0:00	1:16	16.3/35	—	21/29	—	383	—	393	—	16	195	8.2	23.7	54
Aug. 27 22:46		6/9	—	7/8	—	130	—	110	—	16	720	9.9	19	34
Sept. 27 15:37	—	12/24.4	—	19.3/28.2	3.2/4.2	140	—	150	13	4	219	3.2	22.3	41
Sept. 28 15:10		2.1/4.3	—	3.4/4.7	—	120	—	75	—	25	170.8	2	0.8	1.5
Sept. 28 19:17		3/5.9	—	4.3/5.5	—	33	—	42	—	20	110	0.6	15	17
Sept. 28 17:59		2.5/4.8	—	2.9/3.8	—	60	—	45	—	25	134	1.2	10.5	17
Sept. 29 18:40		13/26	—	13/12	—	100	—	95	—	17	25	0.3	7	24
Sept. 29 20:20	20:37	6/13	—	12/10	—	50	—	105	—	14	37	0.3	12	21
Oct. 1 17:58		20/26.6	4/2.7	22/12.6	6-Apr	150	10	140	10	10	85	17	24	36
Oct 0.1 17:58		20.3/28.6	5.4/3	20.1/26.5	3.8/6.9	150	10	140	10	10	85	1	17	24
Oct.2 06:59		13/17	—	11/14	2.6/4	70	—	60	15	15	25	−0.6	13	25
Oct.2 9:33		12/15	—	8.2/11	4/6.4	90	—	20	25	12	37	0	19	45
Oct.28 10:30		16.2/29	—	11.6/15.2	—	370	—	339	—	33	85.4	−1.4	0.5	2.3
Nov.29 18:00		3.6/7.1	3.4/7.5	—	—	81	203	—	—	33	85.4	1.18	1.23	43

In columns 7–10, we show the duration of TGE observed by all 4 mentioned above detectors; all durations are calculated from the start of the enhancement of count rate until its recovery to pre-TGE value. In the 11-th column we show the distance to lightning flash (if any) estimated by the EFM-100 electric mill; in the 12-th column- distance to the cloud base calculated from outside temperature and dew point; in the13-th – outside temperature. And in last 2 columns - the maximal negative strength of the near-surface electrostatic field measured during TGE and amplitude of electrostatic field changes.

**Figure 7:**
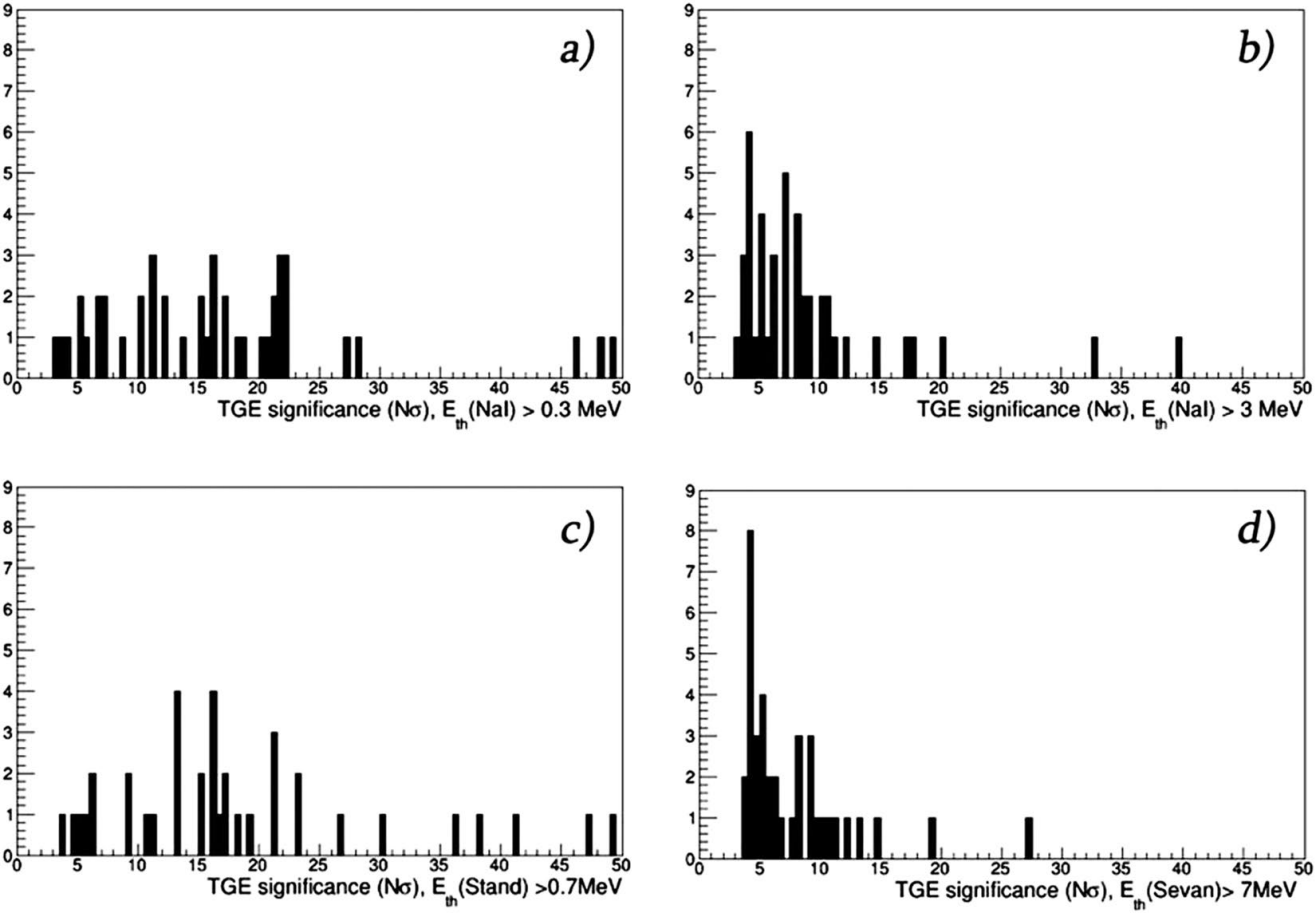
Statistical significance of TGE events containing HEP (44 selected events).

In Fig. 7 we show TGE significances calculated for different particle detectors. Obviously, detectors with lower energy threshold demonstrate highest significances.

**Figure 8:**
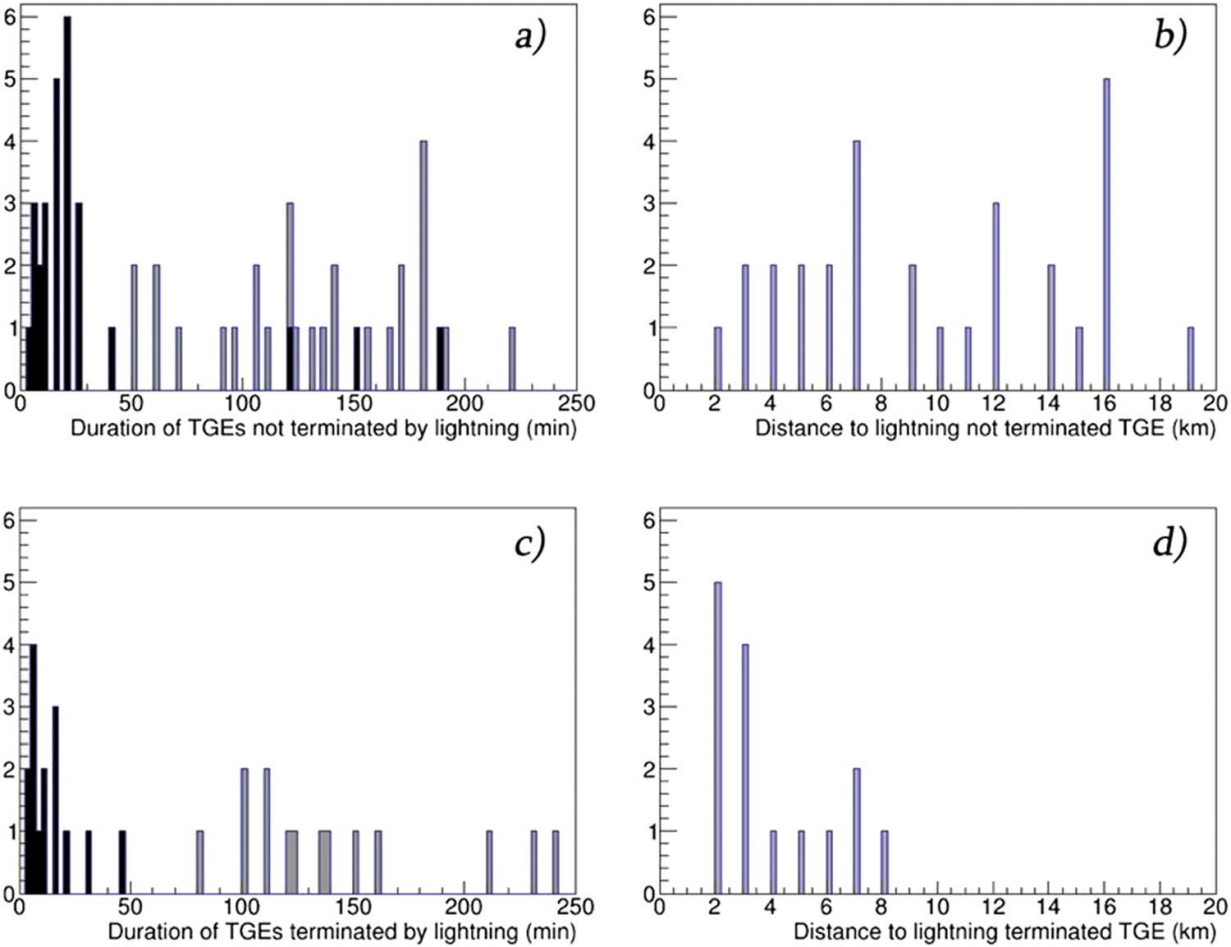
Duration (**a**,**c**) and distances (**b**,**d**) of TGEs terminated and not terminated by the lightning flash. Black –measured by NaI detector with E_th_ > 3 and gray- with E_th_ > 0.3.

In Fig. 8 we demonstrate the duration of TGE events and distance to the lightning flash that terminates TGE. In Fig. 8a,c by bold black we denote TGE events with HEP, by gray – without HEP. Apparently, events containing HEP are shorter in duration, because the probability of lightning is higher.

In Fig. 8b,d we show the distance to lightning flash for both kinds of TGE events. Only nearby lightning flashes (<10 km) terminate the particle flux.

**Figure 9:**
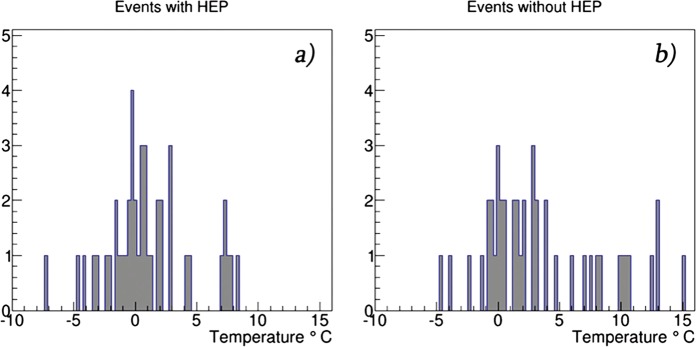
Outside temperature during TGE events with and without HEP.

In Fig. 9 we show the distribution of outside temperature for events containing HEP (9a) and without HEP (9b). Most of largest TGEs occurred when temperature is with in −3–+3 °C.

**Figure 10:**
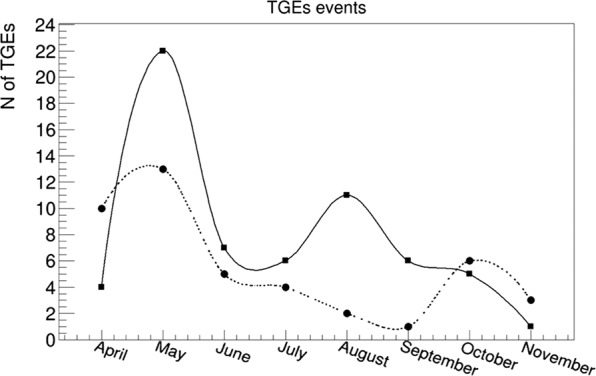
Distribution of TGEs by months. Solid line - TGEs without HEP; dotted line – with HEP.

In Fig. 10 we can see that the frequency of both kinds of TGE strongly peaked in May, when the temperature fluctuates around 0 °C and clouds are very low above surface. In June – July number of TGE declines to recover for TGEs containing HEP in August (start of autumn on Aragats), and in October for TGEs without HEP.

**Figure 11:**
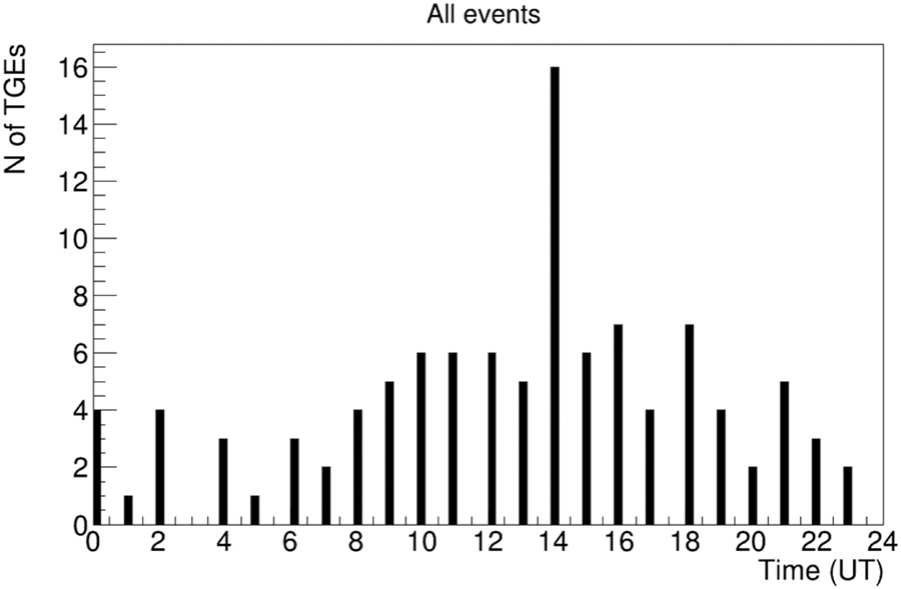
TGE “daily wave”; frequencies of TGE occurrence during the day time.

In Fig. 11 we show the daily frequencies of TGE occurrence. Maximal frequency was observed at 18–19 local time (UT + 4).

## Discussion

We publish the first TGE event catalog. TGE events are very rare and usually, in publications, only one-two observed events are analyzed and discussed. For the first time, we present the whole collection of TGE collection gathered in an year. The natural electron accelerator in the clouds above Aragats station operates continuously providing more than 100 TGEs. All TGEs were analyzed and classified according to the presence or absence of high-energy particles. We present the distribution of the TGE events by months of the year and by hours of the day. The maximal frequency of TGEs occurred in May and araund 14:00 UT (18:00 local time). Strong TGEs happened mostly when the outside temperature is in the (−3–3) C° limits. Only lightning flashes within 10 km can terminate TGE.

Based on our analysis, we can outline new foundlings made in 2017 and confirm our previous conclusions from the decade of observations. Most important founding based on analysis of 2017 data is observation and description of the *long-lasting TGE*. Flux of high-energy particles from the avalanches reaches detectors on earth’s surface and originates bursts of particles. High-energy part of TGE is extending few minutes and usually sharply completed by a nearby lightning flash. Atmospheric discharges that occurred within 10 km, decrease field within the dipole, and terminate the acceleration of leptons to high energies. The long-lasting part of TGE is connected with Compton scattered gamma rays from remote avalanches and with bremsstrahlung emission of electrons gaining additional energy from the intracloud electric fields (MOS process^42^). The gamma radiation from the Radon daughters brought by rain also can contribute to the low energy part of TGE. Mentioned results confirm our statement about “radioactive” thunderclouds^36^. Raw data that was summarized in Tables 1 and 2 is available via the ADEI interactive WEB platform; slides of each-month analysis of TGE data are located in CRD seminars site (http://www.crd.yerphi.am/Slide).

## Methods

**Figure 12:**
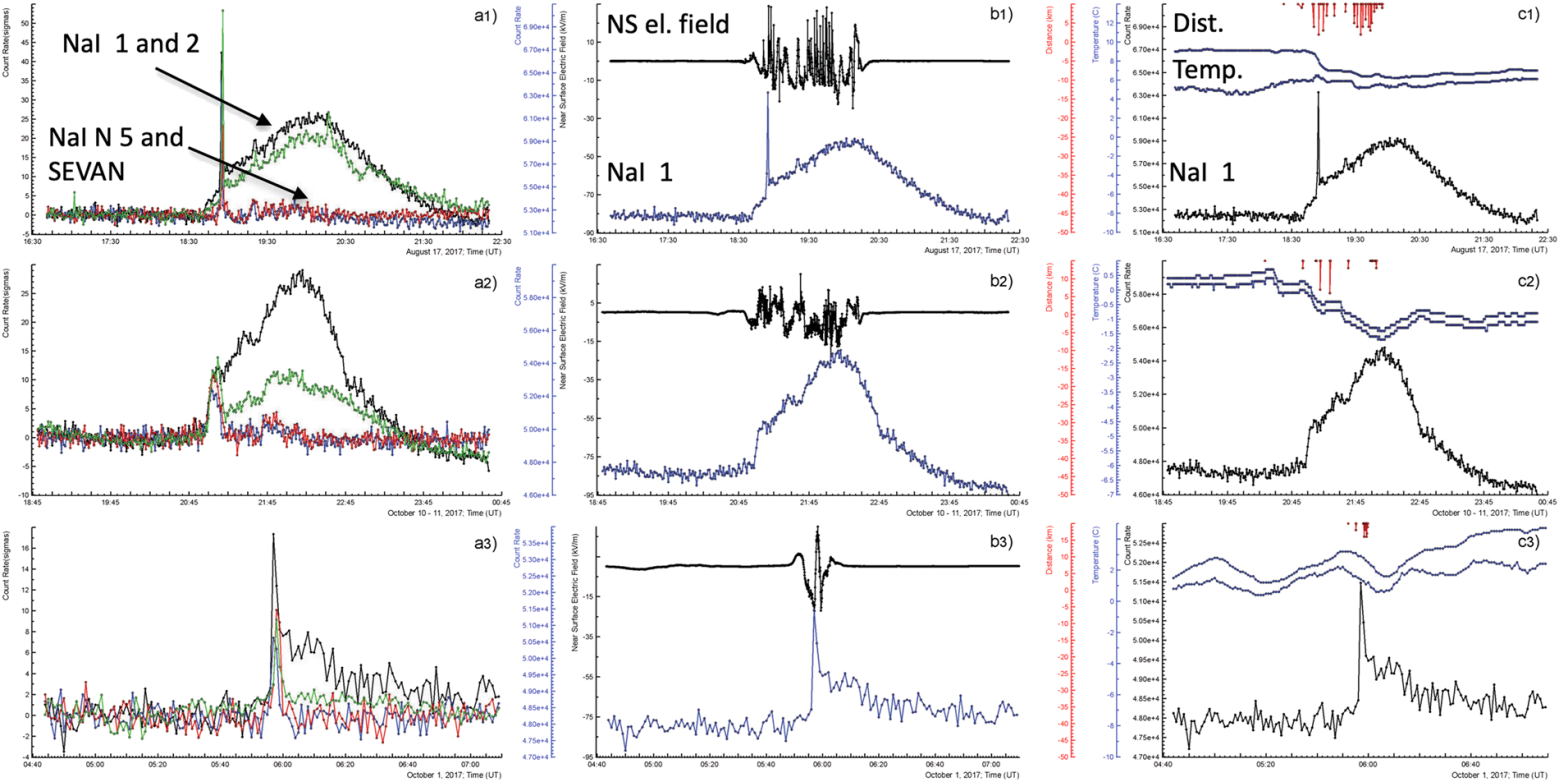
Visualization of the information on TGE observed in 2017 and posted in Tables 1 and 2.

The relationship between time-series of all measured geophysical parameters and elementary particle fluxes can be immediately evaluated using an advanced multidimensional visualization system *ADEI (Advanced Data Extraction Infrastructure)*. ADEI is a WEB data analysis platform to handle large amounts of data stored for a long time and assessable for users worldwide. The overall time interval of measurements is ~ 20 years, and the frequency of data stream from particle detectors now reaches hundreds of KHz. With our analysing system, a catalog of TGE events registered in 2017 for two broad classes of events was compiled. The summary Tables 1 and 2 show several key characteristics of the TGE, and associated meteorological data. In Fig. 12 we show frames visualizing several data analysis options for three selected TGE events occurred on August 17, first line; October 11, second line; and October 1, third line.

In the frames a1, a2, a3 we show the long lasting TGEs measured by particle detectors with energy thresholds equal to 0.3 MeV (NaI detectors N 1 and 2), 5 MeV (NaI detector N 5), and 7 MeV (upper scintillator of SEVAN detector). We can see the short peaks (with large intensities) that corresponds to RRE avalanches in the cloud. Particles with energies of tens of MeV born in these avalanches register all detectors. The long-lasting, low energy part register particle detectors with low energy threshold only. To visualize all very different count rates in one and the same frame we use p-values instead of absolute values of count rates. In frames b1, b2, b3 we show relation of the same TGEs (detector NaI N 1, count rates are in the absolute numbers) to disturbances of the near surface electric field (NS el. field). All TGEs are related to the large disturbances reaching-20kV/m. The strong electric field in the thunderclouds originates electron – gamma ray avalanches, in which electrons are accelerated and multiplying.

In frames c1, c2, c3 we show the relation of meteorological parameters to TGEs (NaI detector N 1). In the middle of frames, we locate outside temperature and dew point (Temp). The rough estimate of cloud base height made with these parameters proves a rather close location of thunderclouds on Aragats (50–200 m) in Summer-Autumn season^36^. The close lightning flashes (2–5 km) from detector site prove the strong electric field above Aragats station.
